# Insulin-like Growth Factor-1 (IGF-1): Demographic, Clinical and Laboratory Data in 120 Consecutive Adult Patients with Thalassaemia Major

**DOI:** 10.4084/MJHID.2014.074

**Published:** 2014-11-01

**Authors:** Vincenzo De Sanctis, Ashraf T Soliman, Giancarlo Candini, Mohamed Yassin, Giuseppe Raiola, Maria Concetta Galati, Rania Elalaily, Heba Elsedfy, Nicos Skordis, Piernicola Garofalo, Salvatore Anastasi, Saveria Campisi, Mehran Karimi, Christos Kattamis, Duran Canatan, Yurdanur Kilinc, Praveen Sobti, Bernadette Fiscina, Mohamed El Kholy

**Affiliations:** 1 Pediatric and Adolescent Outpatient Clinic, Quisisana Hospital, Ferrara, Italy; 2 Department of Pediatrics, Division of Endocrinology, Hamad General Hospital Doha, Qatar; 3 Department of Medical Physics, St. Anna Hospital, Ferrara, Italy; 4 Department of Hematology, Alamal Hospital, Hamad Medical Center, Doha, Qatar; 5 Department of Paediatrics, Pugliese-Ciaccio Hospital, Catanzaro, Italy; 6 Department of Haematology, Thalassaemia and Prenatal Diagnosis Regional Center, Pugliese-Ciaccio Hospital, Catanzaro, Italy; 7 Department of Primary Health Care (PHC), Doha, Qatar; 8 Department of Pediatrics, Ain Shams University, Cairo, Egypt; 9 St. George’s University Medical School at the University of Nicosia, Cyprus; 10 Endocrine Unit, Villa Sofia –Cervello Hospital, Palermo, Italy; 11 Department of Thalassaemia, Garibaldi Hospital, Catania, Italy; 12 Unit for the Diagnosis and Treatment of Thalassaemia, Umberto I Hospital, Siracusa, Italy; 13 Hematology Research Center, Shiraz University of Medical Sciences, Shiraz, Iran; 14 First Department of Paediatrics, University of Athens, Athens, Greece; 15 Director of Hemoglobinopathy Diagnosis Center and President of Mediterranean Blood Diseases Foundation, Antalya, Turkey; 16 Department of Pediatric Hematology, Faculty of Medicine, University of Çukurova, Adana, Turkey; 17 Professor of Pediatrics, Christian Medical College and Hospital, Ludhiana, India; 18 Department of Pediatrics, NYU School of Medicine, New York, USA

## Abstract

**Introduction:**

IGF-1 deficiency in TM patients in children and adolescents has been attributed to chronic anemia and hypoxia, chronic liver disease, iron overload and other associated endocrinopathies, e.g. growth hormone deficiency (GHD). Few data are available in the literature regarding adult TM patients and growth disorders. The aim of this study was to measure IGF-1 values and other clinical data in a large number of adult patients with TM to evaluate the possible relationships between them.

**Patients and Methods:**

A cohort of 120 adult patients with TM was studied for plasma levels of IGF-1. Plasma total IGF-1 was determined by chemiluminescent immunometric assay (CLIA) method. In eleven patients (3 females) the GH response during glucagon stimulation test (GST) was also evaluated.

**Results:**

Fifty percent of patients (33 males and 27 females) had IGF-1 levels <- 2 SDs below normative values for healthy subjects matched for age and sex. In these patients endocrine complications and elevations of aminotransferases (ALT) were more common compared to TM patients with IGF1 > -2SDs. In multivariate regression analyses, height, weight, BMI, serum ferritin, ALT, HCV serology and left ventricular ejection fraction (LVEF) were not significantly related to IGF-1, but a significant correlation was found in females between HCV-RNA positivity and IGF-1, ALT and serum ferritin. AGHD was diagnosed in 6 (4 males) out of 11 patients (54.5%) who had glucagon stimulation tests and in 5 out of 8 (62.5%) with IGF-1 <-2SD. The mean age of patients with GHD was 39.3 years (range: 25–49 years, median: 39 years) versus 35.8 years (range: 27–45 years, median: 37.5 years) in non-GHD patients. A positive correlation between GH peak after GST and IGF-1 level was found (r: 0.6409; p: < 0.05).

**Conclusions:**

In 50% of TM patients the IGF-1 levels were 2SDs below average values for healthy individuals. IGF-1 deficiency was more common in TM patients with associated endocrine complications, and a significant correlation was found in HCV-RNA positive females among IGF-1, ALT, and serum ferritin. Further data in a larger group of patients are needed to confirm whether IGF-1 level <-2 SDs may be a potential criterion for additional studies in TM patients. This datum could avoid performing GH stimulation tests in the majority of them.

## Introduction

IGF-1 is a small peptide (molecular weight 7649 Da), that circulates in serum bound to high affinity binding proteins (IGFBPs), mostly in a ternary complex with IGF-binding protein 3 (IGFBP-3 ) and acid-labile subunit (ALS). This ternary complex is a storage form of IGF-1 in serum and has a half-life of several hours. ALS is synthesized under direct control of GH, primarily in hepatocytes. In contrast to pulsatile GH secretion, circulating IGF-1 and IGFBP-3 are stable and reflect the long-term status of GH secretion.^1^,^2^

Although the liver mainly produces IGF-1, every tissue can secrete IGF-1 for autocrine and paracrine purposes.^3^ IGF-1 possesses a large number of activities (anabolic, antioxidant, anti-inflammatory and cytoprotective actions), however, it is partly responsible for systemic GH actions.^4^ IGF-1 deficiency in TM patients in children and adolescents has been attributed to chronic anemia and hypoxia, chronic liver disease, iron overload and other associated endocrinopathies, e.g. growth hormone deficiency (GHD).^5^–^11^

Few data are available in the literature regarding adult TM patients. Therefore, the International Network of Clinicians for Endocrinopathies in Thalassemia and Adolescence Medicine (ICET-A), previously called the International Network on Endocrine Complications in Thalassemia (I-CET), for a better interpretation of IGF-1 values in thalassaemia made the following proposals:

(1) estimate the IGF-1 values in a large number of adult TM patients; (2) conduct the study in a single center, to minimize the considerable variation in the assay methods used; (3) compare the results with those for a reference population of healthy adults; (4) correlate the IGF-1 values of patients with TM with other clinical and laboratory parameters; (5) perform a provocative GH stimulation test with glucagon (GST) in a small group of TM subjects with normal or low IGF -1 levels (below the mean standard reference levels); (6) to review the current literature on AGHD in thalassaemia with the final goal of assisting clinicians in the management of TM patients with GH-IGF-1 axis related disorders.

## Subjects and Methods

From 2009 to 2013, we studied 120 consecutive adult patients with TM followed at our institution Patients with thalassaemia intermedia, cirrhosis, cardiac and renal failure and HIV positivity were excluded from the study.

An extensive medical history, including data on associated complications and current medications, was obtained, and a physical examination (pubertal status, weight, and height) for each patient was performed. Body mass index (BMI) was calculated as the body weight divided by the height squared (Kg/m^2^). A subject was considered overweight when the BMI was between 25 and 29.9 and obese when the BMI was 30 or higher.

All patients were on regular transfusions (mean haemoglobin level 11.5 g/dl) and iron chelation therapy with deferoxamine (64 patients: 30–45 mg/kg body weight, 4–6 days a week by slow subcutaneous infusion by pump, starting in 1977–1978), or oral deferiprone (22 patients: 75 mg/kg body weight daily), or deferiprone plus deferoxamine (25 patients; 75 mg/kg body weight daily and 40 mg/kg body weight, 3 days a week, by slow subcutaneous pump infusion) or oral deferasirox (9 patients: 20–30 mg/kg body weight daily).

The following clinical and laboratory data were also recorded: age at first transfusion, age at start of regular chelation therapy, duration of iron chelation therapy, compliance with treatment and the presence of associated growth and endocrine complications, as previously described.^12^

The compliance was arbitrarily considered as good when the chelation therapy was given 5–6 days a week, moderate four days a week and poor less than three days a week.

Blood samples were drawn in the morning after an overnight fast and at least 2–3 weeks after the last blood transfusion to measure the serum concentrations of IGF-1, free thyroxine (FT4) and thyrotropin (TSH), urea, creatinine, electrolytes, glucose, calcium, phosphate and serum ferritin. As routine or to exclude severe liver pathology or decreased synthetic functions, alanine aminotransferase (ALT), gamma glutamyl transferase (**γ**GT), alkaline phosphatase (ALP), total and direct bilirubin, albumin, prothrombin time (PT) and international normalization ratio (INR), and serologic screening assays for hepatitis C virus seropositivity (HCVab and HCV-RNA ) were also evaluated.

Iron overload was assessed at the beginning of the study, by serum ferritin level. It was arbitrarily categorized as mild, moderate or severe if the levels were <1000 ng/ml, from 1000 to 2000 ng/ml and >2000 ng/ml, respectively.^13^

In eleven patients (3 females) the GH response, after glucagon stimulation test (GST), was evaluated. Blood samples were collected for 3 hours (at baseline, 30, 60, 90, 120, 150 and 180 minutes), because, according to the literature, the majority of GH peaks occur between 120 and 180 minutes (85%). Serum glucose, insulin and GH were measured; a GH peak below three μg/L was defined as severe GHD, according to Gomez et al.^14^

For ethical reasons we used as a reference for normal the IGF-1 values determined in a large population of healthy subjects, reported in the literature^12^ using the same automated chemiluminescence immunoassay system (CLIA). A serum IGF-1 level below -2 SD was considered as deficiency.

Insulin resistance was also calculated in these patients using HOMA-IR (homeostasis model assessment method). Considering HOMA-IR values, the studied population was defined as a. (insulin sensitive) HOMA-IR < 2.24; b. (intermediate) 2.24 – ≤3.59; 3. (insulin resistant) HOMA-IR > 3.59.^16^

### Biochemical, Hormonal and Cardiac Assessment

Fasting blood samples for circulating IGF-1 were collected and stored at −60°C until centrally assayed. Plasma total IGF-1 was determined on EDTA by chemiluminescent immunometric assay (CLIA) method (Nichols Institute Diagnostics, San Juan, CA). The assay was performed after separation of IGF-1 from binding proteins by Liaison® autoanalyser (DiaSorin SpA, Saluggia, Italy). The sensitivity of the test was six ng/ml, whereas the intra- and inter-assay coefficients of variation (CVs) of our in-house pooled serum control sample were 4.8% and 7.1%, respectively.

Serum GH concentrations were measured in duplicate at each time point with commercial solid-phase two-site chemiluminescent immunoassay. The inter- and intra-assay CVs were below 7%.

TSH and FT4 were assessed with an electrochemoluminescence (ECLIA) assay with a normal range of 0.8–1.8 ng/dL for FT4 and 0.5–4.6 mIU/l for TSH. The minimal detectable levels of FT4 and TSH were 0.2 ng/dL, and 0.1 mIU/L, respectively. The inter-assay and intra-assay coefficients of variation of FT4 varied from 5.8% to 6.26%, and from 2.6% to 2.9%, respectively, and those of TSH were from 5.1% to 5.7%, and from 2.2% to 2.9%, respectively.

All biochemical and serologic tests were carried out in accordance with the routine procedures of the central laboratory. Serum ferritin was measured by electrochemiluminescence immunoassay. Reference range values were 30–350 μg/l in males and 15–150 μg/l in females.

Left ventricular ejection fraction (LVEF, %) was measured according to the recommendation of the American Society of Echocardiography, using freeze-frames from two-dimensional directed M-mode echocardiogram.^17^

Associated endocrine complications were defined according to the I-CET position statement published in 2013.^12^

### Ethical Aspects

The study was made in accordance with the provisions of the Declaration of Helsinki. The prospective study was started at the beginning of 2009 by the Coordinator of I-CET (VDS) at the Thalassaemia Centre of Ferrara, and was completed at the end of 2013 at the Quisisana Pediatric and Adolescent Outpatient Clinic of Ferrara. All of the subjects gave their consent to participate in the study.

### Statistical Analysis

Characteristics of the studied patients are reported as mean ± standard deviation (SD), median, number and range. Quantitative variables are reported as medians (range or percentiles) or mean (SD). Statistical significance of the differences between variables was assessed using the unpaired two-tailed

Student’s t test or Wilcoxon test using a software package program. The frequency distributions for age and sex were analyzed using the chi-square test while the multiple regression analysis was conducted using the multiple linear fitting with least squares method.

The distribution analysis of IGF1 values in males and females groups was performed including the following parameters: mean and SD, median, range, kurtosis, skewness, and percentiles.

Fisher’s Exact test was used to calculate the probability value for the relationship between two dichotomous variables.

A p value < 0.05 was considered as significant.

A software program used for the statistical analysis was developed by Dr. Candini (Department of Medical Physics, St. Anna Hospital, Ferrara, Italy) and validated according to Alder and Roesser.^18^

## Results

The demographic, clinical, and laboratory data of the TM study population are presented in Table 1. Rates of infection with hepatitis C are displayed in Table 2.

A statistical comparison between HCV positive and negative TM patients was not done because only 3 TM patients were HCV negative (Table 2). In addition, using a multivariate discriminant analysis the classification error observed between HCV positive and negative patients was 20% and therefore was considered not clinically acceptable.

An abnormal ALT value (>40 U/L) was observed in 51.8% of female TM patients with IGF-1 levels < -2SD and 23 % of patients with IGF-1 levels > -2SD. Similar results were observed in males (48.4% with IGF-1 levels < -2SD and 26.6% IGF-1 levels > -2SD).

Of the 120 patients, 58 (48%) were males and 62 (52%) females, with an age range of 26.0 – 53.2 (median 38) years for females and 20.8 – 51.2 years for males (median 37.05), 64.4 % of the patients were above 35 years of age. The mean body mass index (BMI) was 22.48 ± 3.34 kg/m2.

The mean value of BMI in TM female patients with IGF 1 <-2SD was 23.8 ± 4.3 (range: 18.6 – 40) and in patients with IGF 1 > -2SD was 23.2 ± 3.5 (range: 17.2 – 32.2. The mean value of BMI in TM male patients with IGF 1 <-2SD was 22.3 ± 2.7 (range: 17.4 – 28) and in patients with IGF 1 > -2SD was 22.1 ± 1.7 (range: 18.5 – 23.2).

Seven TM female patients were classified as overweight and three as obese; 5 TM male patients were classified as overweight and none as obese.

Of 63 patients with hypogonadism, 13 (37%) were on stable sex steroid therapy (Table 1). All patients with primary or central hypothyroidism or hypoparathyroidism were receiving levothyroxine or calcium and calcitriol. The type 1 diabetic patients were treated with insulin.

In males with TM, the serum concentrations of IGF-1 ranged between 18.3 and 147.7 ng/ml (mean 68.29 ± 33.5 ng/ml; median 62.5 ng/ml; kurtosis −0.5, skewness 0.57); whereas, in females with TM, IGF-1 ranged between 19.5 and 195.5 ng/ml (mean 76.46 ± 41.84 ng/ml; median 69.05 ng/ml; kurtosis 0.02; skewness 0.87).

**Figure 1:**
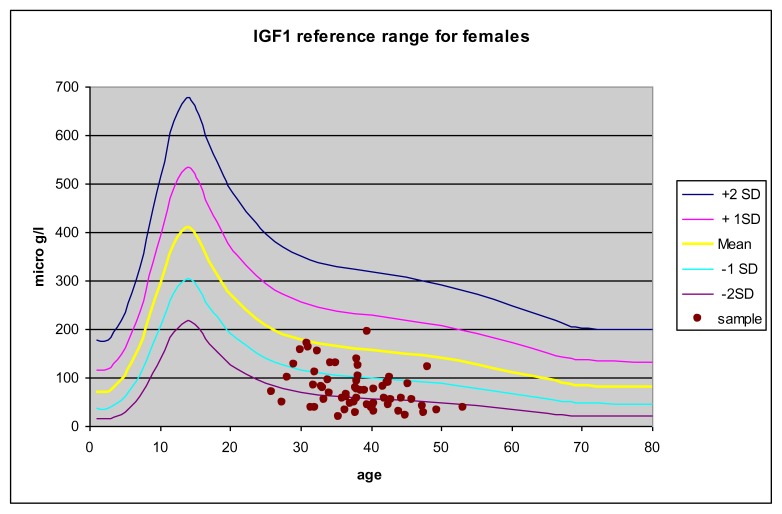
IGF-1 as a function of age in female TM patients compared to the percentile of healthy subjects. Values are reported as mean, ±1 SD and ± 2 SD.

**Figure 2:**
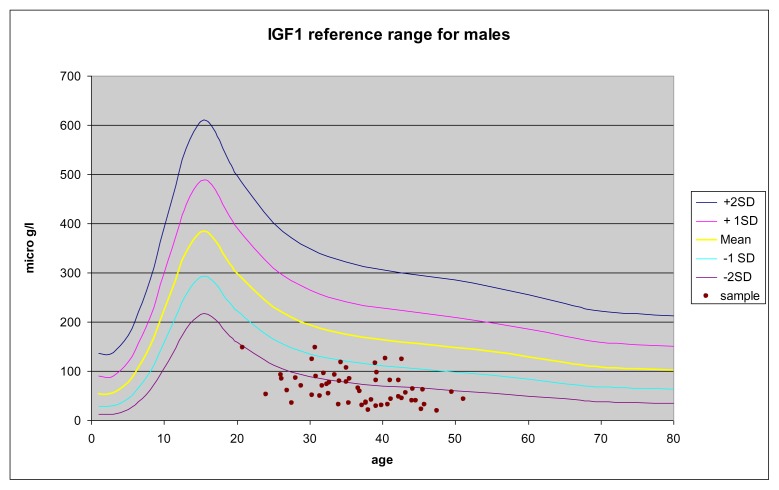
IGF-1 as a function of age in male TM patients compared to the percentile of healthy subjects. Values are reported as mean, ±1 SD and ± 2 SD.

The distribution of IGF-1 values in the two groups of TM patients compared to the percentiles of Brabant et. al 15 are reported in Figures 1 and 2.

No significant differences were observed between IGF-1 values in men and women with TM (t-test: 1.18; p: 0.249). In multivariate regression analyses, height, weight, BMI, serum ferritin, ALT, HCV serology and left ventricular ejection fraction (LVEF) had no significant relationship with IGF-1 levels, but a significant multiple correlation was found in females, with HCV-RNA positivity, between IGF-1, ALT and serum ferritin (r= 0.504, p= 0.043). The simple correlation matrix was as follows: IGF1 vs ALT: r = 0.505 p < 0.05; IGF1 vs serum ferritin: r = − 0.466 p < 0.05; ALT vs serum ferritin: r = − 0.402 p < 0.05.

A statistical comparison of IGF 1 values between HCV positive and negative TM patients was not done because only three patients were HCV negative (Table 2). In addition, using a multivariate discriminant analysis the classification error between patients HCV positive and negative patients was 20% and therefore considered not statistically acceptable.

Analysis of individual IGF-1 levels in TM patients showed that IGF-1 levels were below -2SDs of normal values for healthy individuals^15^ in 60 (50 %) patients (33 males and 27 females).

The demographic, clinical and laboratory features of the TM patients with IGF-1 levels < -2SD below normal are reported in Table 1.

A comparison of clinical and laboratory features of male and female TM patients with IGF-1 < - 2SD and > - 2SD are reported in Tables 3 and 4.

There were significant differences in age, ALT levels, and rates of primary hypothyroidism and insulin-dependent diabetes between female patients with IGF-1 levels <2 SD and those with higher IGF-1 levels; significant differences only for ALT levels and rates of primary hypothyroidism were found in males with IGF-1 <2 SD.

The left ventricular ejection fraction (LVEF) was reduced (< 50%) in 2 patients (1.6%).

AGHD was diagnosed in 6 (4 males) out of 11 patients who had glucagon stimulation tests (54.5 %). The mean age of patients with GHD was 39.3 years (range: 25–49 years) versus 35.8 years (range: 27–45 years) of non-GHD patients.

A positive correlation between GH peak after GST and IGF-1 levels was found (r =0.6409; p: < 0.05). In particular, a low GH peak after GST (range 0.28–1.3 ng/ml) was found in 5 out of 8 patients (aged 25–49 years) with an IGF-1 level below -2SDs (range 16.7 – 52.5 ng/ml) and in 1 out of 2 patients with an IGF-1 level below -1SD (75 ng/ml) (Table 5). No correlation was observed between IGF-1 level, ALT and LVEF.

A HOMA-IR > 3.59 was found in one female TM patient with GHD (Table 5).

In general, in our patients the compliance to treatment was good or satisfactory. A serum ferritin level below 1000 ng/ml was observed in 36 (65.5%) female and 37 (74%) male TM patients. A ferritin level > 2000 ng/ml was observed in 11 (20%) female and 5 (10%) male patients. The remaining patients had a serum ferritin level between 1000 to 2000 ng/ml [11 females (20%) and 8 (16%) males]. However, we did not know with absolute certainty if the patients in the past had a severe iron overload.

## Discussion

In healthy individuals, serum levels of IGF-1 peak at puberty (mean level: 391 ng/ml, between the ages of 14–15 years) and decline with age (mean level: 174 ng/ml at the age of 35 years and 99 ng/ml at the age of 70 years).^15^

GH is the most important factor controlling IGF-1 secretion and concentration. Other factors are also determinant: age, sex, pubertal stage, ethnicity, nutritional function, hepatic status and hormones (sex steroids, thyroxin, and prolactin).^19^–^22^

Very low levels of IGF-1 were found in our adult patients with TM with or without GH deficiency (GHD).^22^–^24^ Of 120 TM patients, 50 % (33 males and 27 females) had IGF-1 levels 2SDs below average values for healthy individuals.^15^

A marked IGF-1 deficiency (IGF1 < -2SD) was more common in TM patients with associated endocrine complications, and multiple significant direct correlations were found in females, with HCV-RNA positivity, among IGF-1, ALT and serum ferritin.

There is increasing evidence that between 8% to 44% of adult patients with TM develop some degree of GHD.^36^–^40^ On GST, GHD was found in 6 out of 11 TM patients studied (54.5%), aged 25–49 years. Of those with GHD, five had IGF < -2SD However, the number of patients was too small to perform a statistical correlation. Similar results were obtained by Soliman et al.^29^ The authors diagnosed IGF-1 deficiency (IGF-1 <-2 SDs) in 20 patients out of 30 (66.6 %). Twelve out of 30 TM patients (40%) had GHD.^41^ Peak GH levels correlated significantly with IGF-1 levels and the height of TM patients expressed in SDS. Neither GH peak nor IGF-1 concentrations were correlated to serum ferritin and liver enzymes.^29^ TM patients with GHD and IGF-1 deficiency also had a significantly lower bone mineral density (BMD) T-score at the lumbar spine compared to patients with normal GH and IGF-1 levels.^29^ These data confirm the role of IGF-1 in the pathophysiology of osteoporosis in addition to prenatal and postnatal body growth.^30^–^37^

An abnormal ALT value (>40 U/L) was observed in 51.8% of our female TM patients with IGF-1 levels < -2SD and 23% of patients with IGF-1 levels > -2SD. Similar results were observed in males (48.4% of TM patients with IGF-1 levels < -2SD and 26.6% of patients with IGF-1 levels > -2SD. Both differences in rates were statistically significant.

In addition to GHD and chronic liver disease, IGF-1 deficiency in patients with TM may be partly due to anemia, iron overload, hypogonadotropic hypogonadism (HH) or its treatment, vitamin D or zinc deficiency.

Soliman et al. have demonstrated the beneficial effect of correcting anemia on increasing serum levels of IGF-1 in children with TM.^24^,^38^ Similarly, correction of other forms of anemia and correction of hypoxia by surgical repair of congenital cyanotic heart lesions have been previously shown to improve IGF-1 secretion, as well as subsequent growth.^39^–^41^ In the study by Soliman et al., however, the TM patients were severely anaemic (7.5 ±1.2 g/dl) before blood transfusion. The increase of mean Hb from 7.5 ± 1.2 g/dl to 9.2 ± 0.8 g/dl was associated with a significant increase in circulating IGF-1 from 53 ± 35 ng/ml to 82.6 ± 39 ng/ml.^24^,^38^

All our TM patients were receiving regular blood transfusions and their mean Hb level was 11.5 g/dl (mean of pre and post-transfusion Hb). Therefore, it is possible that Hb was not a causative factor for the reduced IGF-1 levels found in our patients.

In general, the compliance to chelation treatment was considered good or satisfactory in our patients. A serum ferritin level below 1000 ng/ml was observed in 36 (65.5%) female TM patients and 37 (74%) male patients. However, we did not know with absolute certainty if the patients in the past had severe iron overload.

Seventy-two of our patients (60%) had hypogonadotropic hypogonadism (HH), late-onset hypogonadism (LOH) or secondary amenorrhea (SA); 43 patients had primary or central hypothyroidism (35.8 %); 9 patients (7.5%) had hypoparathyroidism and 15 patients (12.5 %) had insulin-dependent diabetes mellitus. Only one-third (37%) of patients with HH were on sex steroid replacement therapy, but the duration of treatment was variable from patient to patient. Therefore, a statistical analysis between treated, untreated and undertreated TM patients with hypogonadism was not done.

It is well known that oestradiol inhibits IGF1 synthesis in the liver by inducing suppressor of cytokine signalling 3 (SOCS3), which inhibits GH stimulated signal transduction.^42^ On the other hand, testosterone (T) not only enhances hepatic IGF1 synthesis, but also alters the sensitivity of the pituitary gland to negative-feedback regulation of GH secretion, leading to an increase of GH and IGF-1 levels.^43^

Recently, it has been shown that vitamin D increases circulating IGF1 in adults and serum IGF-1 levels are significantly correlated with serum zinc. 44,45 Unfortunately, zinc and vitamin D levels were not assessed because, in the original protocol, prepared in 1998, these variables were not included.

Another well recognised effect of low IGF-1 is the increased risk for developing insulin resistance in humans.^46^ IGF-1 improves insulin resistance both in type 2 diabetes and subjects with more severe insulin resistance.

HOMA–IR was assessed in only eleven patients (all of them had an IGF 1 level < - 1 or -2 SDs) studied for GH response to glucagon stimulation test. None of them had insulin resistance.

Further studies, however, are needed in patients with TM because the prevalence of diabetes varies from 6.4% to 14.1%^47^ and both insulin resistance and decreased insulin secretion contribute to the development of DM.^47^,^48^

In conclusion, although many efforts have been made to explore and define the management of TM patients from the endocrinological standpoint, some important questions still remain.

An IGF-1 level <-2 SDs may be a potential criterion for screening of TM patients for GHD, to avoid performing GH stimulation tests in the majority of them. Nevertheless, further data in a larger group of patients are needed to confirm this finding.

The dysfunction of the GH-IGF-1 axis has significant clinical implications, considering that GHD might contribute to the decline of several tissue functions. IGF-1 is a key peptide involved in cell growth and protein turnover, acting as the primary mediator of many of the responses regulated by GH in tissues.

We believe that the role of liver iron overload in IGF-1 deficiency should be further evaluated by MRI assessment of liver iron concentration (LIC) and not with serum ferritin levels. In addition, the role of chronic active hepatitis C and liver function impairment necessitates more extensive studies.

Finally, given the high prevalence of hormonal deficiencies and the non-specificity of clinical signs and symptoms, a systematic annual endocrine referral is recommended in TM patients. It is advisable to define multidisciplinary cost-effective protocols in which first-line specialists order baseline pituitary function tests and endocrinologists do the clinical evaluation, interpret the hormonal results and evaluate the possible related complications.

## Figures and Tables

**Table 1:** Demographic, clinical and laboratory features of a study population of 120 adults with TM and in 60 TM patients with IGF-1 level < -2SD based on reference values of healthy individuals matched for age and sex (Ref. 12)

	Total study population of TM patients	TM patients with IGF-1 level < -2SD	Total study population vs patients with IGF-1 level < -2SD
Patient number	120	60	
Females/Males	62/58	27/33	
Mean age (years) Females/Males	38.5±5.6/37.5 ± 6.3 P = 0.18 n.s.	40.8±5.5/37.9 ± 7.1 P = 0.09 n.s.	P = 0.003/P = 0.27 n.s.
Mean final height (cm) Females/Males	156.0± 6.9/165.3 ±7.0 P < 0.001	156.1±7.0/165.4±6.2 P < 0.001	P = 0.32 n.s./P = 0.39 n.s.
* ALT (U/L) Females/Males	44.3 ± 39.5/49.0 ± 43.5 P = 0.56 n.s.	53.4±45.4/56.3±49.3 P = 0.32 n.s.	P = 0.02/P = 0.10 n.s.
** Serum ferritin (ng/ml) Females/Males	1106.2 ± 1093.0/855.5 ± 760.2 P = 0.19 n.s.	1126.5±828.3/965.3±878.0 P = 0.09 n.s.	P = 0.12 n.s./P = 0.32 n.s.
Hypogonadotropic hypogonadism (HH) Females/Males	34 (54.8%)/29 (50%)	13 (48.1%)/13 (39.3 %)	=
Late onset hypogonadism in males (LOH)	5 (20.8%)	5 (15.1 %)	=
Secondary amenorrhea (SA)	4 (14.2%)	5 (18.5 %)	=
Primary Hypothyroidism Females/Males	16 (25.8%)/7 (12%)	8 (13.3 %)/16 (26.6 %)	=
Secondary Hypothyroidism Females/Males	13 (20.9%)/7 (12%)	0/0	=
Insulin dependent diabetes Females/Males	11(17.7%)/4 (6.8%)	11 (18.3 %)/3 (5%)	=
Hypoparathyroidism Females/Males	6 (9.6%)/3 (5.1%)	4 (6.6%)/3 (5 %)	=
Adrenal insufficiency Females/Males	0/0	0/0	=

* Normal alanine aminotransferase (ALT) level: ≤ 40 U/L

** The reference serum ferritin range values are 30–350 μg/l in males and 15–150 μg/l in females

**Table 2:** Prevalence of thalassaemia major patients infected with HCV enrolled in the study

	MALES	No.	%	FEMALES	No.	%
**HCV-Ab**	Positive	46	95.8	Positive	52	98.1
**HCV-Ab**	Negative	2	4.2	Negative	1	1.9
**HCV-RNA**	Positive	25	52.1	Positive	25	47.2
**HCV-RNA**	Negative	23	47.9	Negative	28	52.8

**Table 3:** Comparison of clinical and laboratory features of female TM patients with IGF-1 < - 2SD and > - 2SD of reference values of healthy individuals (Ref.^12^)

	IGF-1 > - 2SDs	IGF-1 <- 2SDs	p
**Mean age (years)**	36.3 ± 4.9	40.8±5.5	< 0.01
**Mean final height (cm)**	155.9 ± 6.8	156.1±7.0	n.s.
**ALT (U/L)**	34.8 ± 30.3	53.4±45.4	< 0.05
**EF %**	65.5 ± 5.0	64.7± 6.4	n.s.
**Serum ferritin (ng/ml)**	1085.1±1330.7	1126.5±828.3	n.s.
**Hypogonadotropic hypogonadism (no. +/− cases)**	12 ( 52%)	13 (48.1%)	n.s.
**Primary hypothyroidism (no. +/− cases)**	0 %	26.6%	<0.01
**Insulin-dependent diabetes mellitus (no. +/− cases)**	1 (4,1 %)	11 (18.3 %)	<0.01
**Hypoparathyroidism ( no. +/− cases)**	0 %	4 ( 6.6% )	n.s.

**Table 4:** Comparison of clinical and laboratory features of male TM patients with IGF-1 < - 2SD and > - 2SD of reference values of healthy individuals (Ref.^12^)

	IGF-1 > - 2SDs	IGF-1 <- 2SDs	p
**Mean age (years)**	36.6±4.1	37.9±7.1	n.s.
**Mean final height (cm)**	165.2± 8.8	165.4±6.2	n.s.
**ALT (U/L)**	32.9±19.5	56.3±49.3	< 0.05
**EF %**	63.3±4.1	62.5±5.5	n.s.
**Serum ferritin (ng/ml)**	614.1±295.9	965.3±878.0	n.s.
**Hypogonadotropic hypogonadism ( no. +/− cases)**	12 ( 34.2%)	13 (39.3 %)	n.s.
**Primary hypothyroidism ( no. +/− cases)**	0 %	16 (26.6 %)	<0.01
**Insulin-dependent diabetes mellitus ( no. +/− cases)**	1 (4.1%)	3 (5%)	n.s.
**Hypoparathyroidism ( no. +/− cases)**	0%	3 ( 5 %)	n.s.

**Table 5:** Growth hormone (GH) peak (μg/L) after glucagon stimulation test (GST) given i.m., insulin-growth factor-1 (IGF-1), alanine aminotransferase (ALT) and left ventricular ejection fraction (LVEF) in 11 adult thalassaemia major patients

Age (years)	Sex (M/F)	Serum ferritin (ng/ml) *	GH peak after GST i.m. (μg/L)	IGF-1 (ng/ml) **	ALT (U/L) ***	LVEF (%)	BMI (kg/m^2^)	HOMA-IR
25	M	360	0.28	52.5 (−2 SD : 112)	32	55	20.3	2.1
27	M	4932	5.1	35.1 (−2 SD :112)	135	56	19.6	2.9
45	M	364	0.46	23 (−2 SD: 66)	46	48	26.1	0.8
49	F	610	1.3	33 (−2 SD: 49)	16	69	23	1.2
39	M	356	2.3	28 (−2 SD: 70)	70	63	22	5.2
39	M	485	3.2	37 (−2 SD: 70)	36	63	18.3	0.3
29	M	305	5.9	52.5 (−2 SD: 89)	22	55	23	0.5
39	M	482	1.2	16.7 (−2 SDS: 70)	102	69	21.3	3.4
45	M	644	4.9	63.6 (−2 SD: 66)	28	66	22.4	0.6
37	F	1221	1.5	75 (−2 SD: 63; −1SD: 106)	47	54	20.5	0.8
36	F	940	11.7	160 (−1SD: 106 mean 157)	62	64	21.8	1

* Serum ferritin reference range values are 30–350 μg/l in males and 15–150 μg/l in females.

** In parentheses the – 2 SDs and – 1SDs IGF -1 levels reported by Brabant et al.^12^

*** Normal alanine aminotransferase (ALT) level: ≤ 40 U/L
